# Supplementary material for: Recruitment Patterns and Environmental Sensitivity of Glass Eels of Anguilla japonica in the Yangtze Estuary, China
Source: Biology (Basel). 2024 Jan 20;13(1):56. doi: 10.3390/biology13010056 (PMC10813113; doi:10.3390/biology13010056)
Supplement: Supplementary file 1 [file biology-13-00056-s001.zip › biology-2815150-supplementary.pdf]

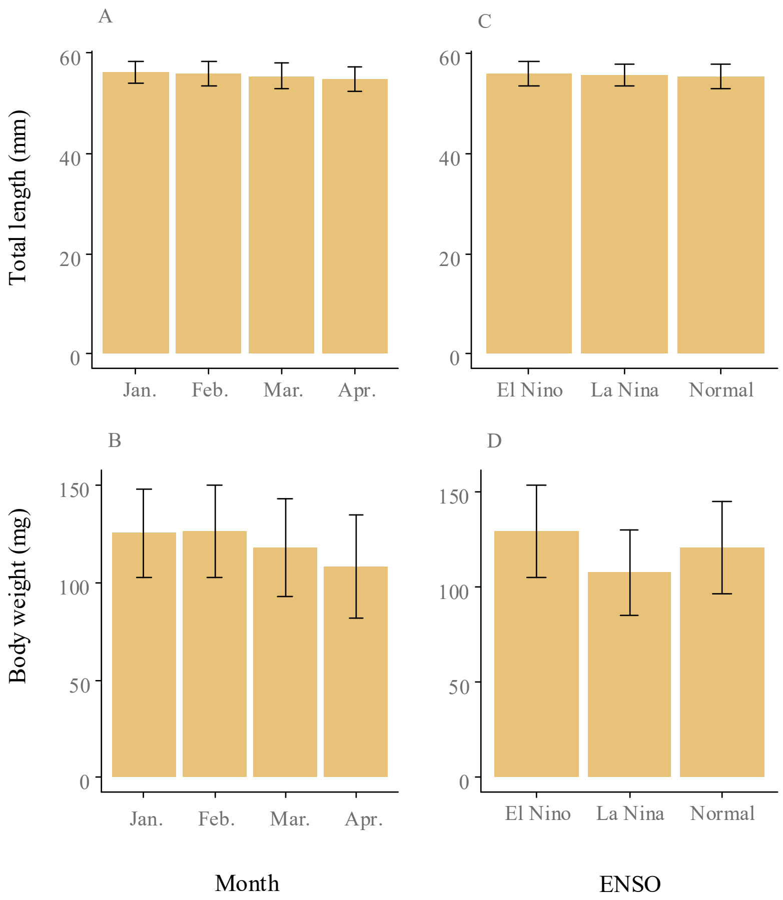

Figure S1. Monthly and ENSO event-based variations in average total length (TL) and body weight (BW) of newly recruited glass eels (stages VA and VB1), with standard deviations. A shows TL by month, B shows BW by month, C shows TL by ENSO event, and D shows BW by ENSO event.

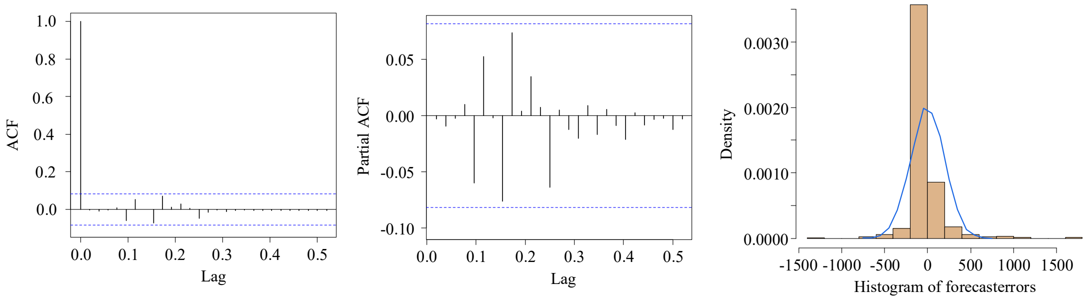

Figure S2. Residuals and forecast errors from the fitted ARIMA(4,0,0)(0,1,1)$_{52}$ model for glass eels weekly catch trends.

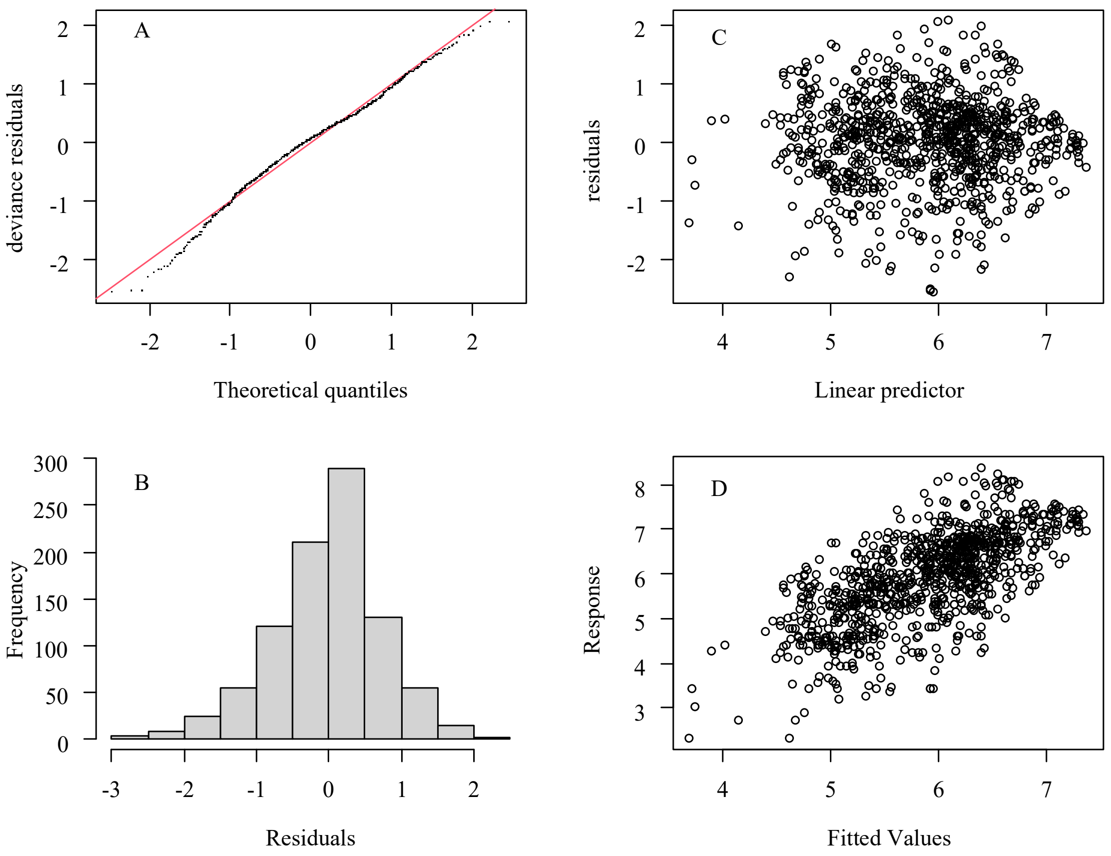

Figure S3. Diagnostic plots for the final generalized additive model (GAM). (A) Quantile-quantile plot of the deviance residuals assessing normality, B) histogram of the standardized residuals to visualize distribution, (C) plot of fitted values against residuals to check homoscedasticity, and (D) plot of fitted values against observed values to evaluate model fit.
